# The Dark Side of the MedPhys Match

**DOI:** 10.1002/acm2.12169

**Published:** 2017-08-30

**Authors:** John A. Antolak, Timothy D. Solberg

## Abstract

This article is related content to the article by Hendrickson et al: https://doi.org/10.1002/acm2.12135

This issue's invited Editorial is provided by John A. Antolak, Chair, AAPM Subcommittee on the Oversight of MedPhys Match and Tim Solberg, Associate Editor of the JACMP. —Michael Mills, JACMP EIC.

Following much debate regarding the appropriate course of medical physics training necessary to enter the field, the AAPM Board of Directors passed professional policy PP 19‐A in 2007: “It is the policy of the AAPM that graduation from an accredited clinical residency program should be a requirement for qualifying for board certification, with an implementation date to be negotiated with the certification boards.” This was despite the fact that few residency programs existed at that time, and counter to the willingness of the American Board of Radiology to accept completion of an approved graduate degree program as an appropriate pathway to board certification. The result was a series of actions and reactions that was in many ways predictable. Residency programs seeking the best candidates often exerted pressure to quickly accept an offer. Candidates occasionally accepted one offer only to rescind it for a later offer. A Gentlemen's Agreement was subsequently implemented but did little to improve the situation. In 2014, the AAPM Board passed professional policy PP 28‐A endorsing a national matching program for medical physics residencies, and together with the Society of Directors of Academic Medical Physics Programs (SDAMPP) made a multiyear commitment to fund the MedPhys Match (MPM).^1^ Among other things, it was hoped that the MPM would improve the recruitment process, eliminate the gamesmanship that existed on the part of both applicants and programs, and create a fair and ethical recruiting environment for applicants and programs.^2^, ^3^

In this issue, Dr. Kristi Hendrickson and colleagues summarize the results of a postmatch survey of physics residency applicants and residency programs that participated in the first two years of the MPM. When Dr. Hendrickson first approached the AAPM Subcommittee on the Oversight of MedPhys Match (SCOMM)^4^ with the idea for doing a survey of programs and applicants, it was readily apparent that her survey would be an excellent way to tell if our profession was actually accomplishing what it had set out to do. Based on their results, we could say that our experience is similar to physicians participating in their respective matching systems, but it is also easy to say that there is plenty of room for improvement.

Somewhat disappointingly, efforts continue on the part of both program and applicants to game the system. While these behaviors might seem rather innocent, they are deceitful and are of almost no benefit to either party. In the words of the authors of the MPM matching algorithm, “both applicants and programs can be advised that trying to get a preferred match by behaving strategically is far more likely to harm than to help them.”^5^

Perhaps more disturbing, the survey results indicate that federal antidiscrimination laws are possibly being violated on a regular basis. As the authors point out, programs rarely do this out of malice, but rather because they are unaware of the illegality and how such practices affect the applicants. Programs need to be aware that federal employment laws cover recruitment for training positions. These laws are in place for very good reasons; therefore, programs should behave accordingly. These behaviors need to be eliminated if at all possible.

The authors provide excellent advice on creating more objective rank lists by reducing or eliminating the influence of postinterview communication, and programs and applicants can both apply this to their rank lists. Finally, their template for interview participants is a good model for emphasizing correct legal and ethical behavior by programs. It is very important that programs model this behavior for applicants; doing so will encourage them to also behave ethically.

As a closing note, it would seem that the desire to seek an advantageous position in any situation is a common characteristic of human nature. Given that, it is prudent to carefully evaluate potential ramifications of any initiative to preempt inevitable shortcomings.

Opinions expressed in this editorial belong to the authors and are not intended to imply endorsement by JACMP, AAPM, SDAMPP, or the authors' employers.
